# Supplementary material for: Identification of photoperiod-induced specific miRNAs in the adrenal glands of Sunite sheep (Ovis aries)
Source: Front Vet Sci. 2022 Jul 22;9:888207. doi: 10.3389/fvets.2022.888207 (PMC9354845; doi:10.3389/fvets.2022.888207)
Supplement: Supplementary file 1 [file Data_Sheet_1.ZIP › Supplementary file/miRDeep2 file.zip!/miRDeep2 file/Know_Structure/SPLP42A1/oar-mir-218a.pdf]

miRBase precursor : oar-mir-218a
Total read count : 44674
oar-miR-218a read count : 44673
remaining reads : 1

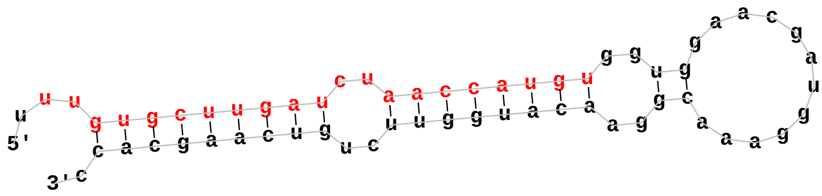

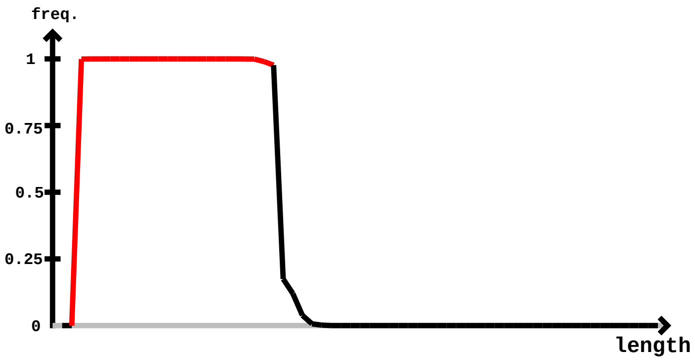

oar-miR-218a

```
5'- uuugugcuugaucuaaccauguggguggaacgauggaaacggaacaugguucugucaagcacc   -3'    exp
    ...(((((((((..((((((((..((............))..))))))))..))))))))).    reads      mm        sample
    uuugugcuugaucuaaccaugu........................................    2          0         seq
    .uugugcuugaucuaacc............................................    10         0         seq
    .uugugcuugaucuaacA............................................    1          1         seq
    .uugugcuugaucuaacca...........................................    24         0         seq
    .uugugcuugaucuaaccau..........................................    412        0         seq
    .uugugcuugaucuaacAau..........................................    1          1         seq
    .uugugcuugaucuaacAaug.........................................    2          1         seq
    .uugugcuugaucuaaccaug.........................................    600        0         seq
    .uugugcuugaucuaacAaugu........................................    104        1         seq
    .uugugcuugaucuaaccaugu........................................    35700      0         seq
    .uugugcuugaucuaaccaugug.......................................    2430       0         seq
    .uugugcuugaucuaaccaugugg......................................    3652       0         seq
    .uugugcuugaucuaaccauguggu.....................................    1417       0         seq
    .uugugcuugaucuaaccauguggug....................................    18         0         seq
    .uugugcuugaucuaaccaugugguU....................................    197        1         seq
    .uugugcuugaucuaaccauguggugg...................................    1          0         seq
    .uugugcuugaucuaaccaugugguUg...................................    72         1         seq
    ..ugugcuugaucuaaccaugu........................................    9          0         seq
    ..ugugcuugaucuaaccaugugg......................................    13         0         seq
    ..ugugcuugaucuaaccauguggu.....................................    3          0         seq
    ..ugugcuugaucuaaccaugugguU....................................    1          1         seq
    ..ugugcuugaucuaaccaugugguUg...................................    1          1         seq
    ...gugcuugaucuaaccaugu........................................    1          0         seq
    ....ugcuugaucuaaccaugugguUg...................................    1          1         seq
    ......cuugaucuaaccaugugg......................................    1          0         seq
    ...........................................caugguucugucaagcac.    1          0         seq
```
